# General practice pharmacists’ implementation of advanced clinical assessment skills: a qualitative study of behavioural determinants

**DOI:** 10.1007/s11096-022-01484-7

**Published:** 2022-10-10

**Authors:** Gordon F. Rushworth, Tesnime Jebara, Antonella Pia Tonna, Ian Rudd, Fiona Stewart, Ronald MacVicar, Scott Cunningham

**Affiliations:** 1 School of Pharmacy and Life Sciences, Robert Gordon University, Aberdeen, AB10 7GJ UK; 2 Highland & Islands Pharmacy Education & Research, NHS Highland, Inverness, IV2 3JH UK; 3 NHS Highland, Old Perth Road, Inverness, IV2 3JH UK; 4 NHS Education for Scotland, 2 Central Quay, 89 Hydepark Street, Glasgow, G3 8BW UK

**Keywords:** Advanced pharmacist practitioner, Advanced practice, Behaviours, General practice, Pharmacy

## Abstract

**Background:**

The role of General Practice Clinical Pharmacists is becoming more clinically complex. Some are undertaking courses to develop their skillsets.

**Aim:**

To explore potential behavioural determinants influencing the implementation of skills gained from Advanced Clinical Examination and Assessment courses by General Practice Clinical Pharmacists.

**Method:**

This study used a qualitative methodology with theoretical underpinning. General Practice Clinical Pharmacists in the Scottish National Health Service, having completed an Advanced Clinical Examination and Assessment course, were invited for online dyadic (paired) interviews. Informed written consent was obtained. The interview schedule was developed using the Theoretical Domains Framework and piloted. Interviews were recorded, transcribed verbatim and analysed using a framework analysis. Ethics approval was obtained.

**Results:**

Seven dyadic interviews were conducted. These included fourteen pharmacist participants from eight Health Boards. Three main themes were identified: 1. Factors influencing implementation of advanced clinical skills by pharmacists; 2. Social and environmental influences affecting opportunities for pharmacists in advanced clinical roles; 3. Perceptions of pharmacist professional identity for advanced practice roles. Nine sub-themes provided a depth of insight including; participants reporting courses allowed clinically autonomous practice; participants shared frustration around social and environmental factors limiting implementation opportunities; participants expressed a need for clarification of professional identify/roles within current contractual mechanisms to allow them to fully implement the skills gained.

**Conclusion:**

This work identified numerous behavioural determinants related to implementation of advanced clinical skills by pharmacists in general practice. Policy, and review of implementation strategies are urgently required to best utilise pharmacists with these skills.

**Supplementary Information:**

The online version contains supplementary material available at 10.1007/s11096-022-01484-7.

## Impact statements

- Advanced Clinical Examination & Assessment (ACE) courses and the skills gained increase General Practice Clinical Pharmacist (GPCPs) confidence in relation to implementation of knowledge, skills and behaviours required to deliver advanced pharmacist practice.
- ACE courses would seem key to development of a versatile Advanced Pharmacist Practitioner (APP) workforce in General Practice with potentially significant impacts for delivery of primary care services.
- While ACE courses provide skills acquisition for advanced clinical assessment, training programmes are needed to triangulate and embed these skills and so support transition to autonomous APP-level practice.
- Policy, and review of implementation strategies are urgently required to ensure pharmacists that have gained advanced clinical assessment skills can provide advanced clinical care to patients in support of existing healthcare services.

## Introduction

The role of qualified pharmacists continues to evolve and expand. To support and sustain progression of practice there is an obvious need to simultaneously develop educational programmes and governance structures to ensure safe and effective care delivery. A recent global survey of country members of the International Pharmaceutical Federation (FIP) reported that advanced pharmacy practice frameworks were in use, or are being developed, in 58% (28/48) of those countries [1]. Despite this, there continues to be high levels of variance as to what would constitute advanced pharmacist practice, even within individual countries [2].

Legislation offers qualified pharmacists the opportunity to function as prescribers in certain parts of the world—in particular; some provinces of Canada [3], United Kingdom (UK), some states of the United States of America (USA) [4] and South Africa [5]. The degree of prescribing autonomy and independence which pharmacists are offered across the world varies in the extreme—from no prescribing rights, through supplementary/complementary prescribing, to independent autonomous prescribing. Within the UK, from 2026 onwards, all newly qualified pharmacists will have legal independent prescribing rights at point of registration [6]. Therefore, in the UK, the right to prescribe itself will not be considered advanced practice. Supporting this concept, the Royal Pharmaceutical Society (RPS) have included independent prescribing in their recently published Post-Registration Foundation Curriculum which sets the standard for entry-level pharmacists in the UK [7].

Advanced pharmacist practice, therefore, is something different, expanding beyond prescribing. Forsyth & Rushworth have set out an opinion of what constitutes an advanced pharmacist role [8]. Central to this is the ability to make autonomous prescribing decisions where there is risk and uncertainty and where there is a limited evidence base. They go on to note that the advanced pharmacist should be able to act upon their own clinical assessment findings and investigate, diagnose, prescribe (where necessary) and manage patients through full episodes of care. The standards expected at this level of practice are set out within the RPS Core Advanced [9] and Consultant [10] Curricula.

If patients and the public are to be assured of the quality of the prescribing pharmacist clinician workforce, then Governments, Regulators, service providers and educational bodies need to consider how to equip pharmacists with the necessary additional clinical skillsets. The Chief Nursing Officer for the Scottish Government has written policy to support the requirements for working as an Advanced Nurse Practitioner (ANP) in a variety of care settings [11, 12]. As part of the educational governance for these roles, all ANPs are required to complete a Master’s level advanced course [13]. Advanced Clinical Examination & Assessment (ACE) courses are taught as modules within these MSc programmes and teach advanced clinical history taking and clinical examination of various physiological systems i.e.: cardiovascular; respiratory; gastrointestinal; musculoskeletal; neurological; ear, nose & throat; and ophthalmology. ACE courses also teach advanced clinical decision-making, such that practitioners can act on the finding of their clinical history and examination. ACE courses are available from numerous Higher Education Institutions.

At present, advanced clinical assessment skills training is absent from most Scottish undergraduate and postgraduate pharmacy curricula and currently limited to ad hoc pharmacist experiences. Therefore, the effect of exposure to this type of training is currently poorly understood within pharmacy. The style of patient interaction within the general practice setting lends itself to autonomous clinical practice. Consultations tend to be had on a one-to-one basis, and while the wider team opinion can always be sought, it is imperative that clinicians working in primary care are able to function autonomously within this environment. General Practice Clinical Pharmacists (GPCPs) have been rolled out across the National Health Service (NHS) in Scotland to support clinical care delivery [14]. Some of these GPCPs have opted to undertake the ACE courses described above to support their transition to advanced level practice but, as yet, there has been no formal exploration of the potential behavioural determinants influencing implementation of advanced skills.

### Aim

To explore potential behavioural determinants influencing the implementation of skills gained from ACE courses by GPCPs.

### Ethics approval

Ethics approval was granted by the Robert Gordon University School of Pharmacy and Life Sciences ethics review committee (Approval Number: S283; 23/Nov/2020). West of Scotland NHS Research Ethics Committee confirmed NHS ethics was not required.

## Method

The research was undertaken according to an interpretivist philosophy using qualitative research methodology.

### Setting

General [Family] Practice (GP) within NHS Scotland.

### Sampling and recruitment

Pharmacists working in Scottish GP practices providing patient facing/clinical services as independent prescribers, having at any time previously undertaken an ACE course, were eligible to participate and included in the study. Secondary care-based pharmacists providing ‘outreach’ services to GP practices were excluded.

There is, to the knowledge of the project team, no single collated source of pharmacists that have completed an ACE course in Scotland. To identify GPCPs who have completed an ACE course and to ensure as many eligible participants as possible were included, Chairs or Leads for key groups representing service, education and academia in Scotland [Directors of Pharmacy; Primary Care Pharmacists Leads (SP3A); Schools of Pharmacy; NHS Education for Scotland] were asked to email their groups with the study invite. Those interested in participating were asked to respond via email. All eligible respondents were then emailed the participant information leaflet and consent form to their work emails (TJ). If no response was received within two weeks, a reminder email was sent. If agreeing to participate, a convenient date and time for the interview was set and informed consent obtained (TJ).

### Development of interview schedule

This work was underpinned by the Theoretical Domains Framework (TDF) [15]. This summarises key elements of 33 theories and proposes that determinants of behaviour cluster into 14 domains. It was initially developed for implementation research to explore behaviours related to evidence-based medicine implementation [16]. The interview schedule was developed using the TDF [15] with those domains most relevant to the research aim and questions included (see supplementary material). The TDF was employed in all stages of the research including developing the interview schedule, analysis, and reporting of findings. The interview schedule was reviewed for face and content validity by all research team members including: pharmacists, GPs, academics and educationalists and then piloted prior to use. No changes were made so the results from the pilot were included in the final dataset.

### Data collection

Dyadic interviews were conducted by a trained researcher (TJ) using video conferencing (Zoom Meeting), digitally recorded then transcribed verbatim and checked for accuracy by the research team using repeated listening and review of transcripts to ensure dependability. Data collection ran in the period of June–August 2021. Dyadic interviews were chosen over individual interviews since they allow participants to share and discuss ideas or issues [17]. They also offer more opportunity than focus groups for in-depth exploration of participant experiences, opinions and recommendations, thus enriching the data generated [18]. Participants were allocated at random to dyads—the principal link between participants was their interest in the research topic [19].

### Data analysis

Framework analysis was undertaken using the TDF as the thematic framework following the steps outlined by Ritchie and Spencer [20]. All interviews were coded based on the domains of the TDF before being examined for further sub-themes within each domain. The analysis was performed by at least two independent team members (TJ and either AT, GR, or SC), with any disagreements resolved through discussion. Illustrative quotes were selected through team discussion. Data saturation was tested using the principals of the approach described by Francis et al. [21]. Initially, six interviews were completed and analysed thematically. An additional interview was then conducted and analysed to ensure that no further themes emerged. Data were managed using NVivo software [version 20, QSR International]. The Consolidated Criteria for Reporting Qualitative Studies (COREQ) was followed in reporting findings of this study [22].

## Results

Seven dyadic interviews containing two participants each were conducted with 14 GPCPs. Participants practiced in eight of the 14 Scottish Health Boards covering approximately 74% of the Scottish population including: Greater Glasgow & Clyde; Grampian; Highland; Lanarkshire; Lothian; Orkney; Shetland; and Tayside. The mean age was 41.14 years old, 57% (n = 8) were female, and 74% (n = 10) were > 10 years post-registration. Each dyadic interview was approximately 60 min long. All pharmacists who responded to the invite were interviewed.

Three main themes were identified with a total of nine sub-themes. These are considered in turn below, with provision of illustrative quotes.

### Theme 1: factors influencing implementation of advanced clinical skills by pharmacists

Key sub-themes presented below are linked to the following TDF domains: Knowledge; Skills; Memory attention and decision process; Beliefs about consequences; Behavioural Regulation.

*Continuum of development:* the ACE course allowed participants to build on the knowledge and skills from their prescribing course and increased their self-perceived capability and confidence in implementation of autonomous clinical decision-making.

“I didn’t really feel when I came out of independent prescribing qualification that I was anywhere near as confident as I am now.” **– P4**

*Advanced clinical skills gained:* Participants noted the course completely changed their decision-making matrix in relation to clinical problems.

“Instead of seeing them as their tablets, you see them much more as a person […] just even having started the course, I’m already thinking completely differently.” **– P9**

As a consequence, participants believed that it enabled them to adopt a holistic approach when reviewing patients, allowing them to provide safer care.

“[ACE skills] will allow me to manage patients more autonomously in a more holistic fashion […] will allow me to prescribe in a safer manner as I will be more aware of red flags and differential diagnosis.” **– P9**

*Boundaries of practice:* Participants believed another consequence was that completing an ACE course highlighted their need for continued preceptored multidisciplinary (MDT) support after the course to embed the skills gained in clinical practice.

“The risk of it is that you kind of feel that I’ve done this now [ACE course], I shouldn’t be asking any questions, or I shouldn’t be utilising other people’s knowledge, when actually that’s absolutely not the way to be going.” **– P10**

*Vision for integration:* Participants articulated a factor aligned to regulation of behaviours through describing the need for a clear vision to encourage greater integration of pharmacists with advanced clinical skills to capitalise on the learning and aid implementation in practice.

“[We need] clear vision of how this will be useful to others so we can get support, get buy in.” **– P9**

### Theme 2: social and environmental influences affecting opportunities for pharmacists in advanced clinical roles

Key sub-themes presented below are linked to the following TDF domains: Environmental context & resources; Social influences.

*Limited number of pharmacists in advanced practice roles:* participants described the environmental context and expressed concern around limited numbers of pharmacists undertaking the course and how this might affect personal and wider professional opportunities for implementation of these skills.

“We still aren’t seeing enough pharmacists through it. It’s quite disappointing […] the value is clearly obvious to people who’ve done it, but it’s not permeating through the workforce.” **– P13**

“Nothing’s changed since we’ve completed it so we’re trying to forge our role.” **– P4**

*Course delivery modality:* in relation to resources available, most participants expressed a preference for face-to-face multidisciplinary courses to aid the acquisition of practical skill, which was thought to aid implementation in practice.

“I found the mix quite good […] to learn from each other.” **– P8**

“It’s a course that you’re looking to gain the actual clinical examination skills and that’s something that’s crucial to be doing in a face-to-face capacity.” **– P14**

*Macro, meso, and micro-level socio-institutional influences:* most participants highlighted influences at a wide range of social-institutional levels describing the limited opportunities to utilise the skills learnt as obstructing the implementation. There was an indication that the reasons for this were complicated and multifarious.

“I think the new [primary care - GMS 2018 [23]] contract has probably taken us a slight step backwards because we were really moving into the clinical patient-facing sphere in general practice, and then pharmacotherapy came forward.” **– P2**

“I work in three different practices, one of them is extremely supportive, [...] At another one, I have to go and seek out support, but if I seek it out, they are willing to give it to me. And the third one isn’t supportive. It’s very stressful […] I’m keen and I want to do this.” **– P9**

Limited understanding of what pharmacists’ offer in terms of clinical service delivery following course completion was a commonly reported perception at practice, line management, Health Board and Government level.

“I think health boards have a responsibility […] to try and work […] to get that message out around ‘we have pharmacists with these skills, they are going to be able to do this’.” **– P10**

“I think that is what’s needed, something from higher up, from Scottish Government level to promote pharmacists extended roles and what the benefits will be.” **– P11**

Participants were hopeful that future changes to Government Policy would create a supportive environment for the implementation and embedding of advanced skills.

“Our work is very much aligned with the GP practice contracts. They will be influenced by Scottish Government […] I’d be quite confident that in another two/three years’ time, people will be working in more advanced roles.” **– P12**

Likewise, participants felt that the RPS Advanced and Consultant Curricula may influence the implementation of advanced roles in practice.

“The RPS advanced practice and consultant practice frameworks are coming along so that can be hopefully utilised to help grow that image […] There are lots of people out there that didn’t really know what a pharmacist can do.” **– P10**

Some had concerns about matters related to organisational governance with articulation of concerns around higher clinical risk and higher indemnity costs which could act as a barrier to implementation.

“We need to pay for indemnity and that comes out of the pharmacist’s pay, it’s not paid for by anybody else.” **– P10**

At the micro level, participants indicated that patients were thought to be broadly supportive of new roles and so could potentially act as advocates influencing implementation.

“As long as you can deliver what they need; assessment, history-taking, plan, reassurance, they take that as they would take it from another professional.” **– P13**

### Theme 3: perceptions of pharmacist professional identity for advanced practice roles

Key sub-themes presented below are linked to the following TDF domains: Social/professional role and identity; Beliefs about capabilities; Emotions.

*Pharmacists’ professional identity in advanced practice roles:* most participants expressed frustration over the currently available career options for GPCPs, which did not cater for integration of ACE course skills, leaving pharmacists feeling deflated about the future and alienated.

“We’ve also got a service direction that isn’t necessarily pushing people into a patient-facing clinical role.” **– P13**

“You don’t want to be sitting all day doing acutes and med rec […] you do want to be managing […] complicated, complex patients and using your prescribing skills in a much more patient focused kind of way.” **– P9**

They noted a lack of understanding of their new advanced roles by other healthcare professionals and management, some of which was attributed to the lack of clearly defined roles for advanced pharmacists but affected their ability to implement.

“I’ve completed the course and I know what it means, but I don’t necessarily think that [GPs] know what it means […] They don’t have an appreciation of how they could use me.” **– P6**

“I feel I didn’t have support from immediate line management because they just didn’t understand.” **– P3**

“I feel definitions of what different pharmacists at different levels in the primary care team do hasn’t really been drawn.” **– P4**

Participants felt senior leadership need to define these roles to optimise utilisation of these pharmacist advanced skills.

“There was no definition of what I was supposed to be doing. I was supposed to have a clinical case load, I hadn’t.” **– P1**

“It needs a little bit of drive from the top, and it needs more in the way of organisation.” **– P5**

*Pharmacists’ self-perceptions of advanced practice role:* Linking to TDF domains emotions and beliefs about capabilities—the course was considered to increase pharmacists’ competence and confidence. However, some participants expressed frustration about deskilling and erosion of capability, especially if they were unable to implement skills in a timely manner once qualified.

“I’m getting frustrated because, as the months go, the confidence in your own ability diminishes exponentially.” **– P6**

“Not to be in a position to use [ACE skills] is incredibly frustrating […] People do this because they want to have that clinical patient facing role.” **– P8**

## Discussion

### Statement of key findings

This work provides data on the potential behavioural determinants influencing implementation of ACE course skills in GPCPs. Key findings relate to the three main themes.

#### Factors influencing implementation of advanced clinical skills by pharmacists

 The ACE course allowed participants to develop knowledge and advanced clinical skills capabilities beyond the scope of traditional pharmacist roles. Those able to use the skills in practice, post-course, reported managing patients with a higher degree of clinical autonomy. Participants described the need to be embedded in an MDT environment and for supervised triangulation of clinical skills and decision-making in practice to aid implementation.

#### Social and environmental influences affecting opportunities for pharmacists in advanced clinical roles

Participants expressed frustration about a range of factors that hindered implementation which were out with their control. There was a clear feeling that Government policy was required to support advanced pharmacist practice. To optimise the opportunity for utilisation of pharmacists’ augmented advanced clinical skillsets, there is a need for senior leadership at local and national level to define and normalise these roles.

#### Perceptions of pharmacist professional identity for advanced practice roles

A widespread limited understanding of advanced pharmacist professional identity was seen as a significant barrier to integration and implementation of these roles into current clinical care teams and demotivating in terms of development of advanced practice roles. This has left some of these innovators feeling a degree of alienation within the profession and wider healthcare team.

### Strengths and weaknesses

Strengths of this research included using robust theory-driven qualitative approach at all steps. Similarly, having an MDT research team benefitted development and testing of topic guide, trustworthiness and applicability of the research. GPCP participants came from a spread of diverse geographical areas and health boards, were exposed to different courses and modalities of teaching and were able to give a breadth of views. Multiple techniques were employed to ensure the robustness of the research and enhance its trustworthiness [24]. To address reflexivity in relation to the impact of personal experiences on research outcomes, differing views were reconciled through iterative discussion within the MDT research team and bracketing of views.

Limitations of this research included the small numbers of participants. However, data saturation was reached using an established method [21]. It is also possible, due to the recruitment methods, that some pharmacists may not have been recruited, missing their views. Some aspects of this research may not be directly transferable to other settings or countries, especially those without pharmacist prescribing rights.

### Interpretation

#### Factors influencing implementation of advanced clinical skills by pharmacists

The positive impact of pharmacists developing and implementing advanced clinical skills has been demonstrated in other sectors of practice—notably Emergency Departments (ED). One study found pharmacists with advanced clinical skills training were able to deal with more than seven times the number of ED presentations as pharmacist prescribers without (5202 vs 719, respectively) [25]. The training programme used [26] has broad similarities with the ACE course reported here, allowing pharmacists to operate beyond “traditional” models of care, as “practitioners”; being responsible for whole episodes of holistic care [27]. This blended skillset enables a versatile workforce, optimising what pharmacists can offer patients and the healthcare system in general. Similar to the GPCP cohort, the effectiveness of pharmacists in these advanced roles is positively correlated with the supportiveness of the learning environment [28].

The GPCP cohort in this study identified the need for a similar “wrap-around” supervised structured training programme to facilitate development. Participants asked for supervision post-ACE qualification so they could undertake their skills with clinical supervision to aid transition to more autonomous practice across the spectrum of presentations they would be expected to cover. Preceptorship models should be explored to enable the clinical supervision of pharmacists in these advanced training roles [29]. Furthermore, participants identified that the RPS Advanced Pharmacist Curriculum may be beneficial in setting the standard for advanced practice and consideration should be made to align any future programmes with this [8, 9].

#### Social and environmental influences affecting opportunities for pharmacists in advanced clinical roles

There is an obvious opportunity to development a similar model to that described above for ED, but within general practice. An “Advanced Pharmacist Practitioner” (APP) model could equip general practice with a modern, dynamic and responsive generalist clinician that offers versatility to service. In addition to traditional clinical therapeutics, polypharmacy and medicines management systems work, an APP could clinically cover urgent and emergency presentations and lead on the management of patients with long-term conditions. The skills developed on the ACE course form the prerequisite skill-base required for the APP role, including those skills required to assess and manage undifferentiated generalist presentations across the age and acuity spectrum. In doing so, this would address the concerns many participants had regarding the limited opportunities for pharmacists in advanced roles in general practice, while also establishing this type of advanced practice as a social norm.

Effectiveness of previous capability frameworks to develop advanced pharmacist practice have been limited [30]. Participants spoke of role suppression under the current GMS 2018 (primary care) contract [23]. Organisational, structural, and cultural barriers to the delivery of advanced pharmacist practice in the general practice setting have been reported in the UK before [31]. Critical to the agency of pharmacists to innovate, must be the inclusion of augmented skillsets, as described here, in subsequent Government policy. It is proposed, therefore, that the role of the APP in general practice be defined in Government policy, funded, and a structured training programme be commissioned for their development.

#### Perceptions of pharmacist professional identity for advanced practice roles

Globally, there is variation as to what would, could or should constitute an Advanced Pharmacist model [2, 32]. This lack of clarity makes the discussion and common understanding of a generalised and globally relevant professional role and identify with defined skillset difficult, if not impossible [28]. Participants in this research highlighted a demotivating aspect related to concerns about the professional identity and lack of understanding of pharmacists in advanced roles. This seemed to be an inter and intra-professional issue.

Misalignment between innovative advanced practice and the clinical scope of the current GMS 2018 contract has led to policy alienation within this cohort. Policy alienation has been described as a ‘disconnection’ comprised of two core components: ‘powerlessness’—when workers feel they are unable to input based on their experience, or have no flexibility for implementation; and ‘meaninglessness’—when workers question the value of the policy to [in this case] patients and health services [33]. Policy alienation has been observed within other UK health service contexts too [34].

### Further research

Consideration should be given to how best to develop techniques that link to each of the themes identified to support behavioural change.

Further research is required to inform the design and evaluation of a training programme for APPs which provides preceptored learning post-ACE course to aid implementation of clinical skills in practice. Delivery and content should map to Government policy to deliver an effective product to meet service need.

## Conclusion

This work has identified a number of TDF-linked behavioural determinants related to implementation of advanced clinical skills by pharmacists in GP. ACE courses would seem to equip pharmacists with the prerequisite skills required for APP-level service. Policy, and review of implementation strategies are urgently required to ensure pharmacists with ACE skills can provide advanced clinical care to patients in support of existing healthcare services.

## Supplementary Information

Below is the link to the electronic supplementary material.

Supplementary file1 (DOCX 17 kb)
