# Supplementary material for: General practice pharmacists’ implementation of advanced clinical assessment skills: a qualitative study of behavioural determinants
Source: Int J Clin Pharm. 2022 Oct 10;44(6):1417–24. doi: 10.1007/s11096-022-01484-7 (PMC9718702; doi:10.1007/s11096-022-01484-7)
Supplement: Supplementary file 1 — Supplementary file1 (DOCX 17 kb) [file 11096_2022_1484_MOESM1_ESM.docx]

Appendix 1: Interview Schedule

| **Table 1: Interview Schedule** | | |
| --- | --- | --- |
| **TDF Domain** | **Question** | **Probes** |
| Knowledge,  Skills,  Behavioural Regulation | Generally, what is the extent of completion and use of Advanced Clinical Assessment (ACE) courses among pharmacist prescribers working within General Practice in Scotland | How common is it in your network to complete an ACE course? How many, where, characteristics, geographic spread?  How, if at all, is this changing practice norms?  Do you see the role complementing that of other HCPs? How/how not? |
| Knowledge  Skills  Beliefs about Consequences | How did you use your ACE course knowledge and skills? and can you give a specific example? | What/When/Where/How often and with whom do you do it?  Are there any skills taught that you don't use in your practice?  What do you think about the importance, in this context, of:   - considering individual learning needs and coaching/mentoring: clinical / educational supervision - practice ‘rehearsal’ opportunity: trying new skills in a low-stakes, low-risk environment, lacking direct scrutiny by others |
| Social Influences | In your experience, what are stakeholders (patients, practice managers, GPs and other practice staff) views and experiences of prescribing pharmacists using ACE skills? | What do you think about the importance of:   - taking opportunities to observe, learn from, and share with peers - patients’ expectations, gratitude and appreciation for the pharmacist’s role - clinical reasoning: decision making, risk management, dealing with uncertainty - autonomous practice |
| Environmental Context & Resources | In your experience, what support is required for the initial implementation / (further) development of such services? | What do think about the importance of:   - considering the regulatory context and medical-legal issues, legal responsibilities and accountabilities, indemnity insurance - developing guidance on processes and how to implement ACE skills into day to day activities. - cultural change: dealing with things that go wrong / errors |
| Social/Professional Role and Identity | In your experience, what is the extent of integration of such clinical pharmacists to the multidisciplinary GP team? | What are the characteristics of the clinical workload / role using ACE skills undertaken by such pharmacists?  What have been the enablers to your integration into the MDT?  What do think of the importance of:   - professional Identity – particularly the need to be viewed as - independent clinicians, responsible decision-makers, and interprofessional collaborators - being ‘accepted’ by colleagues and avoiding “turf battles” |
| Beliefs about Capabilities | How has the completion of an ACE course modified your / do you feel it would modify your beliefs, perceptions, and behaviours about clinical practice? | How do you feel completing the ACE course could / has affected your:   - confidence in your own clinical capabilities? - Thoughts about the positive / negative consequences of using ACE skills – any examples? |
| Optimism  Intentions  Goals | How optimistic are you that using ACE skills will become an integral part of practice in the future? | What further, if anything, do you intend to do to develop use of ACE skills in your practice?  Do you have any ambitions / endpoints you want to achieve? |
| Reinforcement  Emotions | What are the barriers and facilitators to the use of ACE skills | What may act as a reward or incentive to using ACE skills? What would help reinforce this as a normal part of the pharmacists’ role?  How would worries / fears of using ACE skills affect you practising?  What do think of the importance of:   - positive reinforcement from others on the role, use of skills and abilities e.g. Scot Gov acceptance/promotion of the role |
